# Determination of freedom-from-rabies for small Indian mongoose populations in the United States Virgin Islands, 2019–2020

**DOI:** 10.1371/journal.pntd.0009536

**Published:** 2021-07-15

**Authors:** A. Springer Browne, Hannah M. Cranford, Clint N. Morgan, James A. Ellison, Are Berentsen, Nicholas Wiese, Alexandra Medley, John Rossow, Leanne Jankelunas, Alan S. McKinley, Claudia D. Lombard, Nicole F. Angeli, Thomas Kelley, Jennifer Valiulus, Bethany Bradford, Valicia J. Burke-France, Cosme J. Harrison, Irene Guendel, Marissa Taylor, Gerard L. Blanchard, Jeffrey B. Doty, David J. Worthington, David Horner, Keith R. Garcia, Joseph Roth, Brett R. Ellis, Kristine M. Bisgard, Ryan Wallace, Esther M. Ellis

**Affiliations:** 1 Epidemic Intelligence Service, Division of Scientific Education and Professional Development, Centers for Disease Control and Prevention, Atlanta, Georgia, United States of America; 2 US Virgin Islands Department of Health, Christiansted, US Virgin Islands, United States of America; 3 Poxvirus and Rabies Branch, Centers for Disease Control and Prevention, Atlanta, Georgia, United States of America; 4 National Wildlife Research Center, APHIS Wildlife Services, United States Department of Agriculture, Fort Collins, Colorado, United States of America; 5 Laboratory Leadership Service, Division of Scientific Education and Professional Development, Centers for Disease Control and Prevention, Atlanta, Georgia, United States of America; 6 Epidemiology Elective Program, Division of Scientific Education and Professional Development, Centers for Disease Control and Prevention, Atlanta, Georgia, United States of America; 7 Carribbean District, APHIS Wildlife Services, United States Department of Agriculture, Auburn, Alabama, United States of America; 8 Sandy Point National Wildlife Refuge, US Fish & Wildlife Service, Fredericksted, US Virgin Islands, United States of America; 9 US Virgin Islands Department of Planning and Natural Resources, Fredericksted, US Virgin Islands, United States of America; 10 National Park Service, St. John, US Virgin Islands, United States of America; 11 St. Croix Environmental Association, Christiansted, US Virgin Islands, United States of America; 12 US Virgin Islands Department of Agriculture, Kingshill, US Virgin Islands, United States of America; 13 Center for Surveillance, Epidemiology, and Laboratory Services, Centers for Disease Control and Prevention, Atlanta, Georgia, United States of America; Faculty of Science, Ain Shams University (ASU), EGYPT

## Abstract

Mongooses, a nonnative species, are a known reservoir of rabies virus in the Caribbean region. A cross-sectional study of mongooses at 41 field sites on the US Virgin Islands of St. Croix, St. John, and St. Thomas captured 312 mongooses (32% capture rate). We determined the absence of rabies virus by antigen testing and rabies virus exposure by antibody testing in mongoose populations on all three islands. USVI is the first Caribbean state to determine freedom-from-rabies for its mongoose populations with a scientifically-led robust cross-sectional study. Ongoing surveillance activities will determine if other domestic and wildlife populations in USVI are rabies-free.

## Author summary

Mongooses in the Caribbean region are known to carry rabies and infect and kill humans with the deadly virus. While many countries in the Caribbean region assume they are rabies-free, there is a lack of scientifically based surveillance. We prospectively designed a statistically valid study to determine that mongoose populations in the US Virgin Islands are rabies-free and pose no risk of transmission to human or animal populations. We are pursuing further surveillance efforts to establish rabies freedom in all wildlife and domestic animal population in the US Virgin Islands.

## Introduction

**Fig 1:**
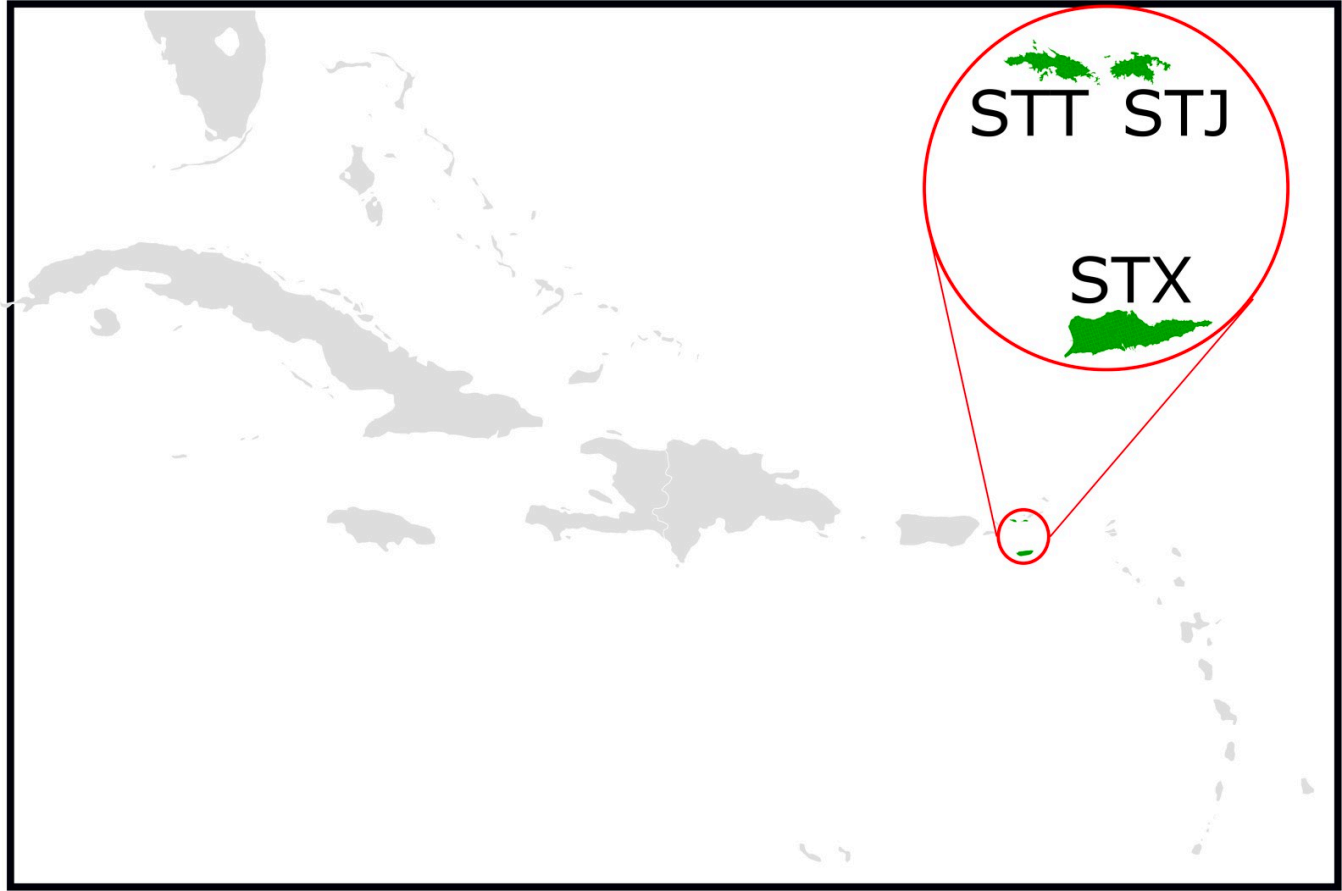
The location of the United States Virgin Islands in the Caribbean region, with the three islands St. Croix (STX), St. John (STJ), and St. Thomas labeled. Map base layers were obtained from https://commons.wikimedia.org/wiki/File:BlankMap-Caribbean.svg and https://catalog.data.gov/de/dataset/tiger-line-shapefile-2017-state-united-states-virgin-islands-current-estate-state-based-shapefi.

Rabies virus infects the nervous system of mammals and without preventive vaccination is over 99% fatal. Although rabies is endemic in 10 Caribbean nations where dogs, mongooses, and bats have been identified as enzootic reservoirs [1], rabies virus has never been detected by existing passive surveillance in the United States Virgin Islands (USVI), a US territory comprising the main islands St. Croix, St. John, and St. Thomas with a total land area of 344 km^2^ and a population of ~100,000 people (**Fig 1**).

In USVI, rabies is a Class A notifiable disease, requiring immediate notification to the local Department of Health for infection in humans or other mammals. Imported domestic mammals (e.g., canine and feline) require rabies vaccination and are monitored by the USVI Department of Agriculture. When rabies is suspected in an animal, brain stem and cerebellum samples from domestic or wild mammals are submitted to the Centers for Disease Control and Prevention (CDC) for rabies testing. Although rabies has never been detected through this existing passive surveillance, fewer than ten samples are tested annually. USVI was presumed to be rabies-free but does not have a scientifically based sylvatic rabies surveillance program to document freedom-from-rabies in USVI wildlife reservoirs (primarily mongooses and bats) (Bethany Bradford, USVI Director of Veterinary Services, pers. comm.). To document absence of rabies, sylvatic rabies surveillance guidelines were developed by the Pan American Health Organization and CaribVET for countries in the Caribbean [2]. Documented freedom-from-rabies could positively impact tourism in USVI, which eliminates costly rabies postexposure prophylaxis administration for residents or visitors bitten by mammals, and justifies strengthening current animal import regulations to keep USVI rabies-free.

Mongooses are an invasive, nonnative pest species in the Caribbean; the small Indian mongoose (*Urva auropunctata*; Syn: *Herpestes auropunctatus*) was historically introduced during the 1870s in the belief that mongooses would combat pests (e.g. rodents) in sugar cane plantations [3]. As opportunistic carnivores, mongooses have thrived in USVI leading to widespread predation of sea turtle nesting sites [4,5] and local endemic species (e.g. St. Croix Lizard, *Ameiva polops*) [6]. The small Indian mongoose became the endemic reservoir of rabies on many Caribbean islands [S1 Video]. In Grenada, the small Indian mongoose is the endemic rabies reservoir; 11.7% were seropositive and 1.7% had rabies virus detected [7]. In Puerto Rico, 39.3% of mongooses sampled were seropositive for rabies virus exposure [8]; rabies virus has also been confirmed in mongooses in Cuba and Dominican Republic [1]. Genomic analyses of the rabies virus suggest that rabies virus was originally introduced to mongoose populations across the Caribbean from canine-lineage viruses: two Puerto Rican rabies variants are closely related to a canine rabies virus variant from the continental United States; Characterization of variants obtained in Grenada suggests it was introduced from an European canine rabies virus >100 years ago [7]; and a Cuban rabies variant is closely related to a Mexican canine rabies variant [9].

The close proximity of Puerto Rico, which has enzootic rabies in its mongoose populations and is approximately 40 miles from USVI, poses a high risk of spillover to the USVI mongoose population [10]. Because rabies is also enzootic on other Caribbean islands, the USVI Department of Health collaborated with local and federal officials to initiate surveillance projects to demonstrate freedom-from-rabies in USVI for domestic mammals and wildlife. These surveillance projects were developed according to guidelines developed by the Pan American Health Organization and CaribVET [2], and the Office International des Epizooties (OIE) Terrestrial Animal Health Code, Standards for Animal Health Surveillance (Chapter 1.4) [11]. We report the findings of a prospective cross-sectional study to determine the absence of rabies in mongoose populations in USVI.

## Methods

### Ethics statement

All animal sampling procedures were approved by the CDC Institutional Animal Care and Use Committee (IACUC), under protocol number 2929DOTMULX-A5; the CDC determined this project was exempt from Human Subject Research protocol review. This IACUC protocol details required field practices, including requirements for anesthesia, and is in accordance with current AVMA guidelines for humane euthanasia [12]. All personnel handling mongooses were vaccinated with one of two FDA-approved rabies vaccines (IMOVAX, RabAvert) within the past year; titer checks were performed on all local collaborators who had been previously vaccinated over a year before field work commenced. Sampling permits were obtained from the National Park Service (Permit #VIIS-2019-SCI-0028), US Fish & Wildlife Service (Sandy Point National Wildlife Refuge Research and Monitoring Special Use Permit #2019–005), and USVI Department of Planning and Natural Resources (Permit #DFW19049U).

### Study design

Our research objective was to determine freedom-from-rabies in mongooses in USVI. In order to detect rabies in the mongoose population in USVI, we assumed a prevalence of 10%–20% rabies exposed mongooses based on known prevalence data from the Caribbean [1,7,8]. The required sample size for presumed level of prevalence of disease in each population is shown in **Table A in S1 Text**, based on Bayesian methods that take into account the sensitivity (100%) and specificity (98.34%) [13] for the Rapid Fluorescent Foci Inhibition Test (RFFIT) on serum samples [14]. Direct fluorescent antibody (DFA) testing of brain stem and cerebellum samples is the gold standard for rabies virus antigen detection (100% sensitivity, 100% specificity) [15]. The RFFIT test detects rabies virus neutralizing antibodies in serum, which indicates if the animal has been exposed to rabies virus antigen; DFA testing uses a fluorescently-labeled anti-rabies antibody to bind to rabies antigen in brain tissue. EpiTools FreeCalc was used to determine the required sample size (http://epitools.ausvet.com.au/content.php?page=FreeCalc2); this method takes into account the population size, presumed prevalence, and the sensitivity and specificity of the screening test to account for increased sampling required with imperfect tests [14]. All laboratory testing was performed by the CDC Rabies Laboratory, a World Organization for Animal Health (OIE) reference laboratory.

**Fig 2:**
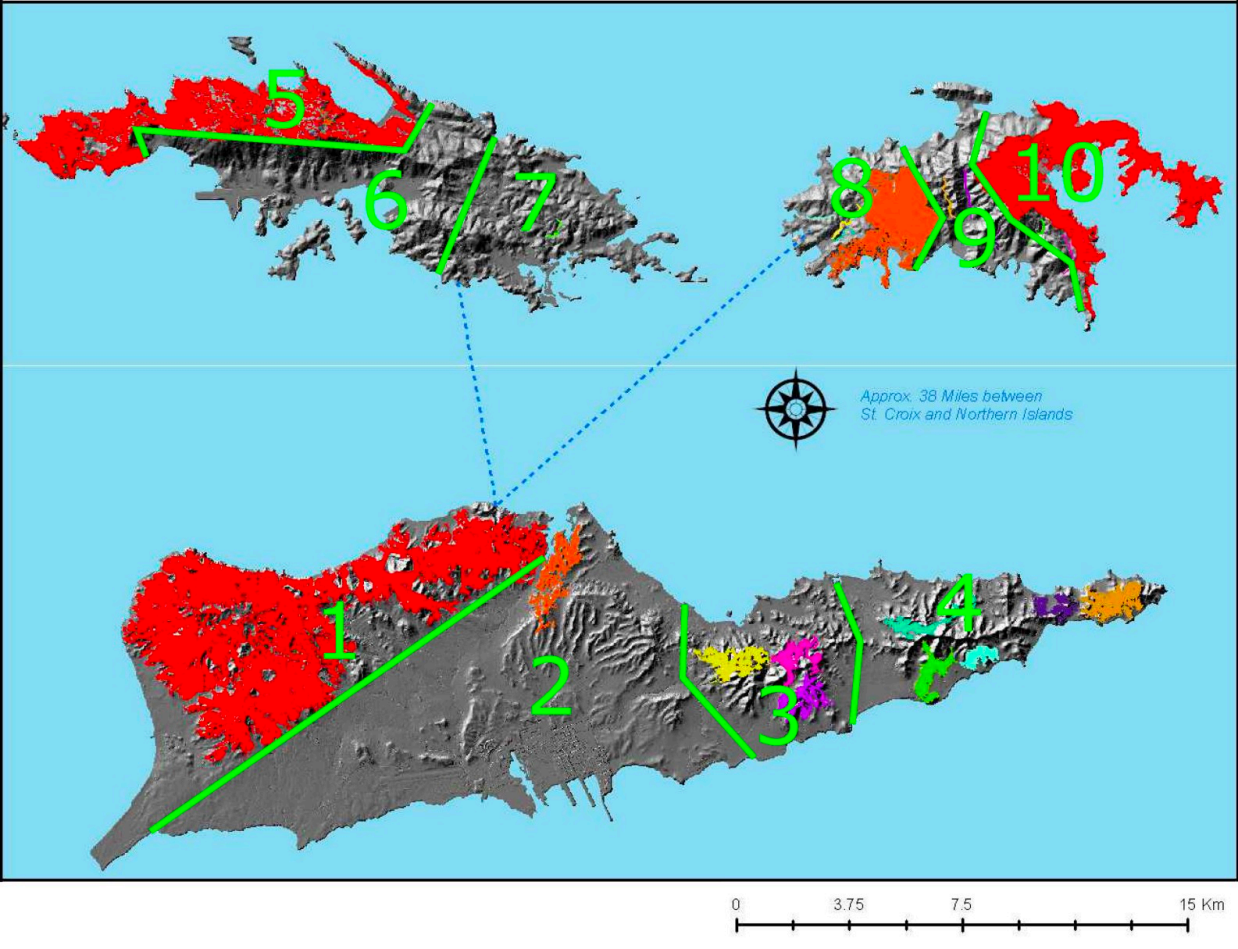
USVI mongoose sampling regions (n = 10) for determination of rabies freedom, with contiguous forest regions highlighted. This figure was adapted from Chakroff, Virgin Islands Department of Agriculture [16].

Ten geographical areas were selected on the three main islands of St. Croix, St. John, and St. Thomas for mongoose sampling based on contiguous forest and ecological areas (**Fig 2**). Based on sample size analyses, a minimum target sampling of 24 mongooses per region was established, with at least two sampling sites selected per region to increase detection sensitivity.

### Sampling methods

Mongooses were sampled during 17 August 2019 to 12 March 2020. Because mongooses are an invasive pest species, they were euthanized before specimen collection; we live-captured mongooses in 20 x 20 x 30 cm cage traps (Tomahawk Trap Co., Hazelhurst, WI, USA) using Libby’s Vienna Sausage as bait. Sex was determined by physical examination, and weight measured using a Pesola spring scale. Blood was collected from the heart and placed in serum separator tubes (BD, 7.5 mL volume); brain stem and cerebellum samples were extracted and placed in a sterile cryovial. Samples were stored on ice in the field; initial processing of serum was done within 12 hours and serum and brain samples were stored in a -80°C freezer. Samples were shipped frozen to the CDC Rabies laboratory for serological testing using RFFIT [17]; antigen testing of brain samples used the DFA test [18]. All mongoose sera were tested using the RFFIT assay for rabies virus neutralizing antibodies (RVNA) with positivity defined as complete rabies virus neutralization at 1:5 dilution (~0.11 IU/ml). For antigen testing, brain impressions were fixed in acetone at -20°C, and RABV antigens were detected by the DFA test, using fluorescein isothiocyanate (FITC)-labelled monoclonal antibody (mAb) conjugate (Fujirebio Diagnostics, Inc., Malvern, PA, USA) as described [19].

### Analyses

Data analysis and map generation were performed with R [20] and ArcGIS desktop version 10.7.1 (ESRI, Redlands, California), and images were edited with Inkscape version 1.0.1 [21].

## Results

**Fig 3:**
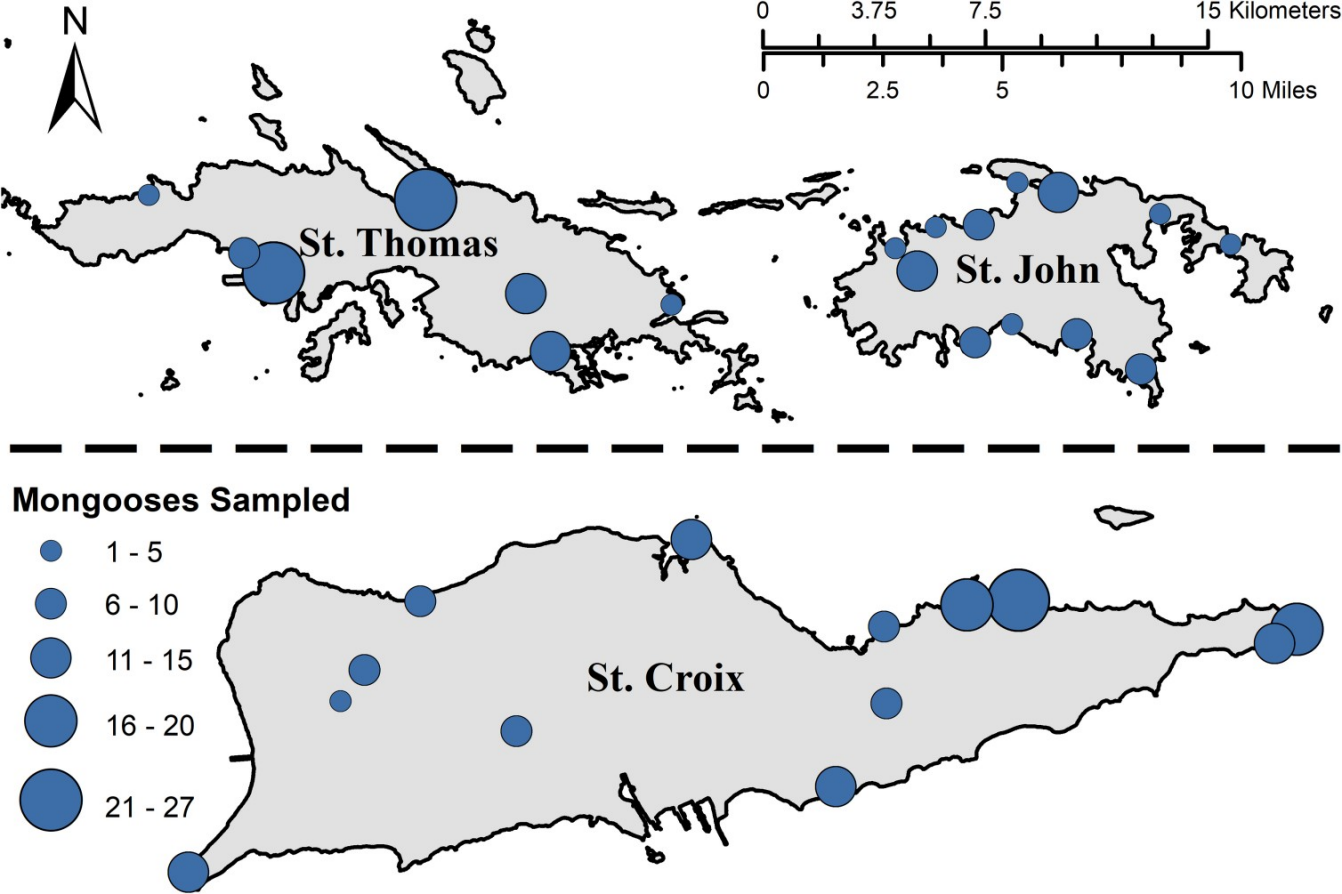
Location and number of small Indian mongooses (*Urva auropunctata*) sampled (n = 312) for rabies antibody and antigen—United States Virgin Islands, 2019–2020. All mongoose serum and brain samples tested negative for rabies antibody by using the rapid fluorescent focus inhibition test, and rabies antigen by using the direct fluorescent antibody test, respectively. Map base layer was obtained from https://www.sciencebase.gov/catalog/item/5ae3f8ede4b0e2c2dd320df8.

During 2019–2020, 312 mongooses were captured and sampled on the islands of St. Croix, St. John, and St. Thomas (**Fig 3** and **Table 1**). All mongooses tested negative for rabies virus neutralizing antibody (n = 300) by RFFIT and negative for rabies virus antigen on brain samples using DFA (n = 296); some samples were not tested due to issues with serum quality (RFFIT) or brain stem and cerebellum sample material (DFA) were not obtained. Based on known rabies prevalence data from the Caribbean [8,22], the sample size obtained from each sampling region equaled or exceeded the requirements for detection of rabies virus in mongoose populations in USVI for either test (**Table B in S1 Text**), and the proportion of mongooses positive for rabies was 0% (95% confidence interval [CI] 0%–0%).

**Table 1:** Location and sex of small Indian mongooses (*Urva auropunctata*) sampled for rabies antigen and antibody testing (n = 312)* —United States Virgin Islands, 2019–2020.

	All sampled (n = 312)			RFFIT tested (n = 300)			DFA tested (n = 296)		
Sex	St. Croix	St. Thomas	St. John	St. Croix	St. Thomas	St. John	St. Croix	St. Thomas	St. John
**Female**	78	46	41	74	44	40	76	46	41
**Male**	73	40	34	71	38	33	60	39	34
**Totals**	151	86	75	145	82	73	136	85	75

*All mongoose serum and brain samples tested negative for rabies antibody by using the rapid fluorescent focus inhibition test (<0.05 IU/ml), and rabies antigen by using the direct fluorescent antibody test, respectively.

**Fig 4:**
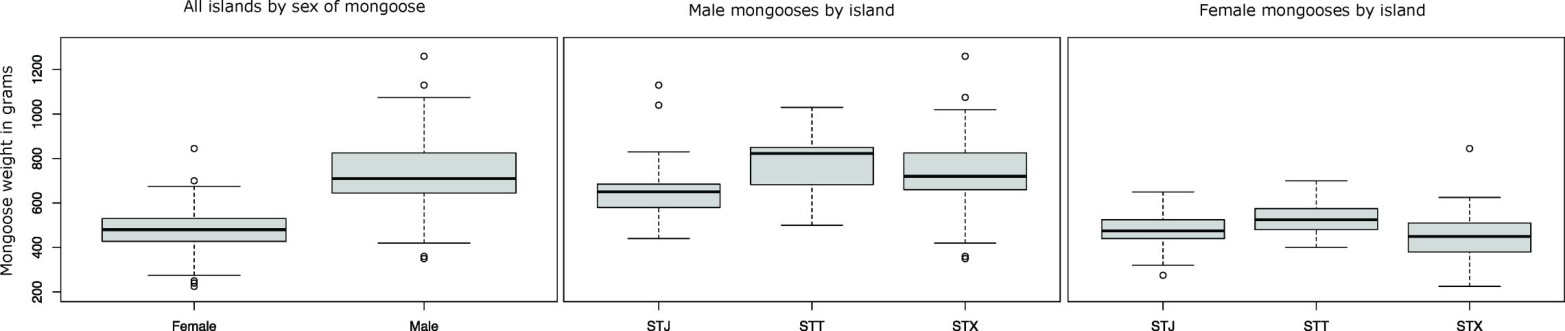
Mongoose (n = 312) weight (grams) comparisons by sex and island*—United States Virgin Islands, 2019–2020, including St. Croix (STX), St. John (STJ), and St. Thomas (STT).

Characteristics of the mongoose population in USVI are shown in **Table 2**. Male mongooses (mean weight 728 g) were significantly larger (p < .0001) than female mongooses (mean weight 481 g) for each island, and mean weights of male and female mongooses was significantly different between islands (p = .003 for male mongooses; p < .0001 for female mongooses) (**Fig 4)**. St. Thomas mongooses were larger than mongooses on the other islands (**Table 2**).

**Table 2:** Mean weight in grams (95% CI) of small Indian mongooses (*Urva auropunctata*) sampled (n = 312)—United States Virgin Islands, 2019–2020.

	Female (n = 165)*	Male (n = 147)*
St. Croix (n = 151)	453g (430–476)	727g (689–765)
St. John (n = 75)	476g (453–499)	664g (617–711)
St. Thomas (n = 86)	533g (512–554)	784g (744–824)
All islands† (n = 312)	481g (467–495)	728g (703–753)

*ANOVA test: p = .003 for male mongooses between islands; p < .0001 for female mongooses between islands

†T-test: p < .000001 for weight difference between male and female mongooses

A total of 886 traps were deployed (median 20 cages deployed daily, range 20–46 cages). Trapping was attempted at 41 field sites (St. Croix: 20, St. Thomas: 13, St. John: 28), with successful capture of at least one mongoose at 32 field sites (**Table B in S1 Text**); 61 field site deployments occurred as some sites were sampled more than one trap-day. Overall, we obtained 31.9% [95% CI 30.1–33.7] capture success per trap-day (St. Croix: 43.3% (95% CI 40.0%–46.6%); St. Thomas: 34.4% (95% CI 30.5%–38.3%); St. John: 19.8% (95% CI 18.1%–21.5%).

## Discussion

This prospective cross-sectional study determined that rabies is not present in mongoose populations on the main islands of USVI (St. Croix, St. John, St. Thomas). This finding is a stark contrast to other Caribbean nations, including Puerto Rico, Cuba, Granada, and the Dominican Republic, where rabies is endemic in mongoose populations [1]. In neighboring Puerto Rico, 39.3% of mongooses sampled were seropositive for rabies exposure [8], while bat serum samples obtained from a single cave in Puerto Rico during 2012–2014 revealed 14/216 samples had positive anti-rabies neutralizing antibodies [23]. Canine rabies has been identified through genomic epidemiology as the source of endemic rabies virus variants of mongooses in the Caribbean [7,9]. USVI has never had a rabies virus detected in canines nor bats, and risk modelling for endemic bat rabies in the Caribbean revealed USVI to be at “low risk” [24]. It is possible that the lack of rabies in mongooses indicates a lack of other endemic rabies reservoirs (e.g. bats, canines) on all three islands.

Ongoing biosecurity and surveillance activities for rabies in USVI is essential, and active sylvatic rabies surveillance in bats and canine populations began in August 2020. Our mongoose rabies active surveillance activities helped to build local public health capacity and collaborations in USVI for zoonotic disease surveillance; increases in trained staff and laboratory capacity will allow for the detection and mitigation of future potential zoonotic disease outbreaks. Our study protocol was disseminated to other Caribbean nations through the Pan American Health Organization to provide a template for rabies surveillance activities throughout the region.

The USVI Department of Agriculture continues importation surveillance and vaccination requirements for domestic mammals. Wildlife importation in cargo ships and shipping containers remains an ongoing concern to prevent introduction of rabid mammals to USVI. In 2019, a live male juvenile raccoon was successfully detected and captured in the St. Thomas container terminal after escaping from a recently imported shipping container from Florida (A. S. McKinley, USDA-WS-Wildlife Services Caribbean Region, pers. comm). Mongooses have also been transported from the Caribbean to Florida through shipping containers in 1978 and 2020 ([25], CDC Rabies team, pers. comm). These translocation events could provide the highest risk of rabid mongoose introduction to these geographically isolated islands. The potential for migration of rabies infected bats from neighboring islands, such as Puerto Rico, is an ongoing concern, and warrant ongoing sylvatic rabies surveillance.

Mongoose populations on USVI maintained similar weights (**Table 2**) as mongooses in Puerto Rico: male mongooses (n = 144) from Puerto Rico had a mean weight of 601 grams (453–964 gram range), and female mongooses (n = 135) had a mean weight of 453 gram (312–680 gram range) [26]. Mongooses are opportunistic scavengers, and the dense human population on St. Thomas with the availability of human food waste, could explain the larger size of both male and female mongooses on this island (**Table 2**).

In sensitive habitats in USVI, mongoose populations are actively managed. During the sea turtle nesting season for sea turtle conservation, mongooses are reduced through intensive periodic trapping and euthanasia; intensive trapping and euthanasia over 5 days on St. John was found to decrease local mongoose populations by 86% [4]. Mongoose predation of sea turtle nests on protected beaches on St. Croix was observed during our sampling, and capture rates were 85% the first day at one heavily predated field site (Prune Bay). In Guadeloupe and Martinique in the Caribbean region, statistical modelling of small Indian mongoose populations revealed mongooses were well established throughout various habitats on these islands and had few or no competition for resources [27]. Because mongooses are well established throughout USVI, eradication is unrealistic and has met with limited success in other island nations [28]. Ecological modelling has predicted that that due to climate change, suitable habitat for mongooses will expand worldwide, to the detriment of local endemic species [29]. Ongoing conservation activities to remove mongooses from sensitive habitats and testing them for rabies will provide vital continued surveillance data to maintain rabies-free status for mongoose populations in USVI.

Limitations of our study included the duration of sampling for this cross-sectional study (approximately 7 months) and the selection of sampling locations. Exposure to tropical conditions (i.e. high heat and humidity), remote sampling locations, and operational and laboratory resources limited the speed that mongooses were captured. However, we were able to obtain the required sample number based on prospective sampling design, obtaining mongoose samples from 32 separate field sites across ten sampling regions. Recent research, using extensive statistical modelling of rabies transmission and persistence for mongooses in the Caribbean region, estimated 34.7% of mongooses in a population would be exposed to rabies virus, were rabies virus present on an island; our study used a conservative estimate of 10% prevalence [30], therefore we exceeded the sampling requirement to determine freedom-from-rabies for mongoose populations on St. Croix, St. John, and St. Thomas islands.

In 2015, the first reported case of human rabies associated with a mongoose bite in North America led to the death of a 54 year-old man in Puerto Rico [31]. Mongoose bites to domestic dogs were also reported to the USVI Department of Health and USVI Department of Agriculture during our study period. Mongooses can pose a public health risk to both humans and domestic animals, and our findings that in 2020–2021, USVI mongooses are rabies-free will benefit residents and visitors to USVI by decreasing mitigation efforts needed following reports of mongoose bites. Ongoing surveillance and removal activities of mongooses, including roadside surveillance of dead mongooses, culling of mongooses as part of sea turtle conservation efforts, and advanced statistical modelling using scenario-tree-modelling approaches to determine the frequency of sampling required and sample number needed to maintain a determination of freedom-from-rabies for mongoose populations, will be used to provide continued assurance of rabies freedom among USVI mongooses. Additional surveillance activities and epidemiological analysis are needed to determine if all domestic mammals and wildlife in USVI are rabies-free, with the goal of providing self-declaration of rabies freedom for USVI to the OIE.

## Supporting information

S1 Video Mongoose bites have been known to transfer rabies virus to humans. (MOV)

S1 Text **Table A:** Sample size required to determine the presence of disease in a given population, based on presumed level of prevalence of disease in that population*. **Table B:** Mongooses sampled by region and sampling location—United States Virgin Islands, 2019–2020.
(DOCX)
