# Supplementary material for: Determination of freedom-from-rabies for small Indian mongoose populations in the United States Virgin Islands, 2019–2020
Source: PLoS Negl Trop Dis. 2021 Jul 15;15(7):e0009536. doi: 10.1371/journal.pntd.0009536 (PMC8282080; doi:10.1371/journal.pntd.0009536)
Supplement: S1 Text — Table A: Sample size required to determine the presence of disease in a given population, based on presumed level of prevalence of disease in that population*. Table B: Mongooses sampled by region and sampling location—United States Virgin Islands, 2019–2020. (DOCX) [file pntd.0009536.s002.docx]

S1 Text: Supporting information

Table A: Sample size required to determine the presence of disease in a given population, based on presumed level of prevalence of disease in that population* (26)

| **Population size** | **10% prevalence** | **15% prevalence** | **20% prevalence** |
| --- | --- | --- | --- |
| 100 | 22 | 16 | 12 |
| 500 | 24 | 17 | 13 |
| 1000 | 24 | 17 | 13 |
| Infinite | 24 | 17 | 13 |

*Antibody test sensitivity 100%; test specificity 98.34%

Table B: Mongoose sampled by region and sampling location — United States Virgin Islands, 2019–2020

*Brain stem and cerebellum sample not obtained from every mongoose sampled

| **Island** | **Region** | **Sampling Location** | **Number of mongooses sampled** |
| --- | --- | --- | --- |
| St. Croix (STX)  (n = 151) | West  (Zone 1)  (n = 31) | Carambola | 8 |
|  |  | Jolly_Hill | 5 |
|  |  | Montpellier | 6 |
|  |  | Sandy_Point | 12* (5/12 serum only) |
|  | Central  (Zone 2)  (n = 30) | Lower_Love | 8 |
|  |  | Haypenny_Beach | 11 |
|  |  | Salt_River | 11 |
|  | East  (Zone 3)  (n = 36) | Altona_Lagoon | 10 |
|  |  | Recovery_Hill | 6 |
|  |  | Southgate | 20* (10/20 serum only) |
|  | Far East  (Zone 4)  (n = 54) | East_End_Bay | 17 |
|  |  | Issacs_Bay | 14 |
|  |  | Prune_Bay | 23 |
| St. Thomas (STT)  (n = 86) | West  (Zone 5)  (n = 29) | Magens_Bay | 27* (1/27 serum only) |
|  |  | Stumpy_Bay | 2 |
|  | Central  (Zone 6)  (n = 28) | Airport | 21 |
|  |  | Brewers_Bay | 7 |
|  | East  (Zone 7)  (n = 29) | Bovoni_Landfill | 11 |
|  |  | Tutu | 14 |
|  |  | Red_Hook_Point | 4 |
| St. John (STJ)  (n = 75) | West  (Zone 8)  (n = 24) | Gifft_Hill_Landfill | 13 |
|  |  | Hawksnest_Beach | 3 |
|  |  | Trunk_Bay | 1 |
|  |  | Western_Reef | 7 |
|  | Central  (Zone 9)  (n = 27) | Cinnamon_Bay | 7 |
|  |  | Francis_Bay | 2 |
|  |  | Lameshur_Bay | 10 |
|  |  | Reef_Bay | 5 |
|  | East  (Zone 10)  (n = 24) | Annaberg_Plantation | 13 |
|  |  | Brown_Bay | 3 |
|  |  | Haulover_Bay | 1 |
|  |  | Salt_Pond | 10 |
